# Computational and phylogenetic validation of nematode horizontal gene transfer

**DOI:** 10.1186/1741-7007-9-9

**Published:** 2011-02-22

**Authors:** Elizabeth H Scholl, David McK Bird

**Affiliations:** 1 Department of Plant Pathology, NC State University, Raleigh, NC 27695, USA; 2 Bioinformatics Research Center, NC State University, Raleigh, NC 27695, USA

## Abstract

Sequencing of expressed genes has shown that nematodes, particularly the plant-parasitic nematodes, have genes purportedly acquired from other kingdoms by horizontal gene transfer. The prevailing orthodoxy is that such transfer has been a driving force in the evolution of niche specificity, and a recent paper in *BMC Evolutionary Biology *that presents a detailed phylogenetic analysis of cellulase genes in the free-living nematode *Pristionchus pacificus *at the species, genus and family levels substantiates this hypothesis.

See research article: http://www.biomedcentral.com/1471-2148/11/13

## The horizontal gene transfer hypothesis

In 1998, Smant and colleagues [1] made a surprising discovery about the cellulases produced by plant-parasitic nematodes (PPNs). The surprise was not just that an animal produced cellulase, as nematode cellulases had previously been demonstrated biochemically [2]. Rather, analyses of the nematode cellulases revealed a phylogenetic incongruence: the deduced protein sequences appeared to be of bacterial origin, whereas the cellulase genes bore all the hallmarks of being eukaryotic. Cloning of genes encoding PPN secretions implicated other candidates as having been acquired from bacteria by horizontal gene transfer (HGT), and a comprehensive computational analysis of expressed sequence tag (EST) data [3] confirmed the widespread occurrence in PPNs of phylogenetically incongruent genes encoding a myriad of functions. The availability of complete PPN genomes [4] has enabled a full reckoning of these genes, and recent work by Danchin and colleagues [5] proposes hypotheses about their specific bacterial origins. The genomic location of the HGT candidates suggested that, once acquired, the genes had undergone reiterative expansion into gene families that presumably exhibit functional diversity [6], although for any given gene and family the 'multiple acquisition versus expansion' hypotheses remain hard to distinguish.

**Figure 1:**
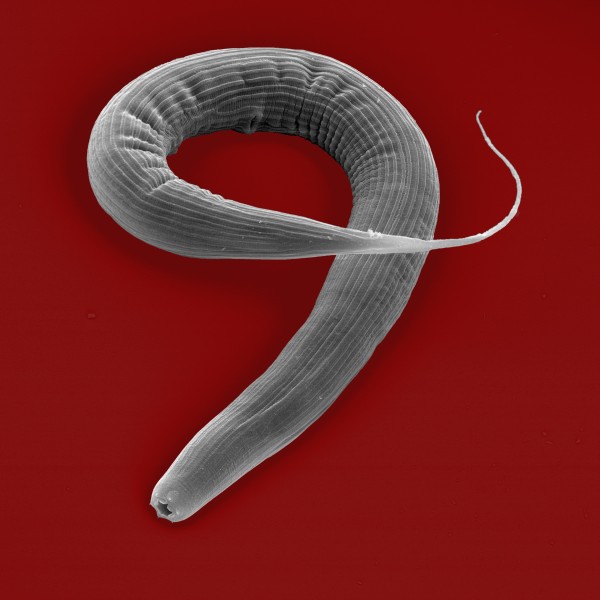
**Scanning electron micrograph of the free-living nematode *Pristionchus pacificus***. Image courtesy of Ralf Sommer, Max Planck Institute for Developmental Biology, Tübingen, Germany.

Part of the appeal of the HGT model for PPN is that many of the functions encoded by these genes can be reconciled with the biology of the nematode. It seems especially obvious that acquisition of genes encoding phytolytic enzymes by a plant parasite would be advantageous. What then should be made of the discovery that the free-living nematode *Pristionchus pacificus * (Figure 1) also encodes cellulolytic enzymes [7]; were these enzymes also acquired by HGT? As they report in a recent paper in *BMC Evolutionary Biology*, Ralf Sommer and colleagues (Mayer *et al. *[8]) set out to investigate four criteria they deemed requisite for successful HGT: that the gene is functionally active; that it is integrated into the host genome; that it displays longevity; and that it shows evidence of having experienced selective pressure.

## Diplogastrid cellulases

By sequencing the transcriptomes of seven *Pristionchus *species as well as three closely related diplogastrid nematodes (*Diplogasteroides magnus*, *Koerneria sudhausi *and *Acrostichus *species), the authors were able to leverage a well-established phylogenetic relationship between the ten species to conduct a series of hypothesis-driven inquiries at the species, genus and family levels of evolutionary relationships [8]. The focus of this work is on a family of cellulase genes thought to have been acquired from an ancestral slime-mold species in an ancient HGT event. The cellulases were identified in all seven *Pristionchus *species and in *K. sudhausi*, but were not found in the remaining two species. Assays confirmed a perfect correlation between the presence of the gene in the transcriptomic data and demonstrable cellulase activity, thus satisfying the first criteria of functional activity of a horizontally acquired gene.

A comparison of the nematode cellulase genes with the cellulase genes in slime molds indicates that amelioration, the process by which the foreign gene assumes the identity of its host, has taken place. Notably, intron size and number in the genes reflect the general gene-structure patterns found within each species. This satisfies the second criterion that the genes be integrated into the host genome after a transfer event. Innovatively, Mayer *et al. *[8] build on the current phylogenetic framework to address the last two features of successful HGT, namely longevity and selective pressure. The 12 *Pristionchus *cellulase ESTs that contained the carbohydrate-binding module at their carboxyl terminus were used in a phylogenetic reconstruction of the genes' history. For 11 of the 12 genes, the topology of the phylogeny was identical with that of the species tree. The observed concordance of the gene history with the species history implies that the gene transfer must have survived multiple speciation events and points to it being ancient. This is a strong indicator of longevity of the cellulases following acquisition by an ancestral species.

## Evolution of *Pristionchus *cellulases

It is in addressing the final question - pertaining to the selective pressures acting on genes that have been horizontally acquired - that the power of this approach is seen. A comparison by Mayer *et al. *[8] of cellulase genes amplified from the genomes of 24 distinct *P. pacificus *isolates revealed an interesting history of the evolution of *Ppa-cel-1*, *Ppa-cel-2 *and *Ppa-cel-3*. Although *Ppa-cel-1 *and *Ppa-cel-2 *were identified in all the isolates, *Ppa-cel-3 *was found only in 16 of the 24 genomes. Sequence-similarity comparisons of the three genes within the isolates suggest that *Ppa-cel-3 *is the result of a recent duplication of *Ppa-cel-2*, and has not yet become fixed in the species. This evidence of gene turnover would seem to be a clear example of active evolutionary pressures on the species. Using empirical Bayesian methods to examine synonymous and nonsynonymous substitutions at specific sites across the gene, a likelihood ratio test indicated significant site-specific positive selection in *Ppa-cel-2*, but not in *Ppa-cel-1 *or *Ppa-cel-3*, thus showing differential selection on the three cellulases within the *P. pacificus *isolates.

This detailed analysis of the evolutionary dynamics of a family of horizontally acquired genes is the most comprehensive to date. By addressing multiple criteria indicative of successful HGT, Mayer *et al. *[8] provide compelling evidence bolstering the hypothesis that HGT is indeed an important component in nematode evolution. Beyond mere scientific curiosity, however, genes acquired by HGT may represent potential targets for the development of novel nematicides or vaccines, both of which are sorely needed. And it would be interesting to know why a bacterivorus nematode encodes cellulase.

## References

[B1] Smant G Stokkermans JPWG Yan Y de Boer JM Baum TJ Wang X Hussey RS Gommers FJ Henrissat B Davis EL Helder J Schots A Bakker J Endogenous cellulases in animals: Isolation of beta-1,4-endoglucanase genes from two species of plant-parasitic cyst nematodes Proc Natl Acad Sci USA 1998 95 4906 4911 10.1073/pnas.95.9.4906 9560201 PMC20186

[B2] Bird AF Downton JS Hawker JS Cellulase secretion by second stage larvae of the root-knot nematode (*Meloidogyne javanica*) Marcellia 1975 38 165 169

[B3] Scholl EH Thorne JL McCarter JP Bird DMcK Horizontally transferred genes in plant-parasitic nematodes: A high-throughput genomic approach Genome Biol 2003 4 R39 10.1186/gb-2003-4-6-r39 12801413 PMC193618

[B4] Bird DMcK Williamson VM Abad P McCarter J Danchin EGJ Castagnone-Sereno P Opperman CH The genomes of root-knot nematodes Annu Rev Phytopathol 2009 47 333 351 10.1146/annurev-phyto-080508-081839 19400640

[B5] Danchin EGJ Rosso MN Vieira P de Almeida-Engler J Coutinho PM Henrissat B Abad P Multiple lateral gene transfers and duplications have promoted plant parasitism ability in nematodes Proc Natl Acad Sci U S A 2010 107 17651 17656 10.1073/pnas.1008486107 20876108 PMC2955110

[B6] Opperman CH Bird DM Williamson VM Rokhsar DH Burke M Cohn J Cromer J Diener S Gajan J Graham S Houfek TD Liu Q Mitros T Schaff J Schaffer R Scholl EH Sosinski BR Thomas VP Windham E Sequence and genetic map of *Meloidogyne hapla*: A compact nematode genome for plant parasitism Proc Natl Acad Sci USA 2008 105 14802 14807 10.1073/pnas.0805946105 18809916 PMC2547418

[B7] Dieterich C Clifton SW Schuster LN Chinwalla A Delehaunty K Dinkelacker I Fulton L Fulton R Godfrey J Minx P Mitreva M Roeseler W Tian H Witte H Yang SP Wilson RK Sommer RJ The *Pristionchus pacificus *genome provides a unique perspective on nematode lifestyle and parasitism Nature Genet 2008 40 1193 1198 10.1038/ng.227 18806794 PMC3816844

[B8] Mayer WE Schuster LN Bartelmes G Dieterich C Sommer RJ Horizontal gene transfer of microbial cellulases into nematode genomes is associated with functional assimilation and gene turnover BMC Evol Biol 2011 11 13 10.1186/1471-2148-11-13 21232122 PMC3032686

